# Processing deficits for familiar and novel faces in patients with left posterior fusiform lesions

**DOI:** 10.1016/j.cortex.2015.02.003

**Published:** 2015-11

**Authors:** Daniel J. Roberts, Matthew A. Lambon Ralph, Esther Kim, Marie-Josephe Tainturier, Pelagie M. Beeson, Steven Z. Rapcsak, Anna M. Woollams

**Affiliations:** a Research Centre in Brain and Behaviour, Liverpool John Moores University, UK; b Neuroscience and Aphasia Research Unit, School of Psychological Sciences, University of Manchester, UK; c Department of Speech Pathology and Audiology, University of Alberta, Canada; d Bilingual Aphasia Lab, School of Psychology, Bangor University, UK; e Department of Speech, Language, and Hearing Sciences, University of Arizona, USA; f Department of Neurology, University of Arizona, USA; g Neurology Section, Southern Arizona VA Health Care System, Tucson, AZ, USA

**Keywords:** Posterior fusiform gyrus, Ventral occipito-temporal cortex, Word recognition, Pure alexia, Face recognition

## Abstract

Pure alexia (PA) arises from damage to the left posterior fusiform gyrus (pFG) and the striking reading disorder that defines this condition has meant that such patients are often cited as evidence for the specialisation of this region to processing of written words. There is, however, an alternative view that suggests this region is devoted to processing of high acuity foveal input, which is particularly salient for complex visual stimuli like letter strings. Previous reports have highlighted disrupted processing of non-linguistic visual stimuli after damage to the left pFG, both for familiar and unfamiliar objects and also for novel faces. This study explored the nature of face processing deficits in patients with left pFG damage. Identification of famous faces was found to be compromised in both expressive and receptive tasks. Discrimination of novel faces was also impaired, particularly for those that varied in terms of second-order spacing information, and this deficit was most apparent for the patients with the more severe reading deficits. Interestingly, discrimination of faces that varied in terms of feature identity was considerably better in these patients and it was performance in this condition that was related to the size of the length effects shown in reading. This finding complements functional imaging studies showing left pFG activation for faces varying only in spacing and frontal activation for faces varying only on features. These results suggest that the sequential part-based processing strategy that promotes the length effect in the reading of these patients also allows them to discriminate between faces on the basis of feature identity, but processing of second-order configural information is most compromised due to their left pFG lesion. This study supports a view in which the left pFG is specialised for processing of high acuity foveal visual information that supports processing of both words and faces.

## 1 Introduction

Humans are highly skilled at visual processing, capable of rapid and accurate identification of a wide range of objects over variations in lighting and viewpoint. Two types of stimuli with which we have considerable experience and expertise are faces and words. Reading is a relatively late-acquired process both in evolutionary and developmental terms (Patterson & Lambon Ralph, 1999), yet it is an essential and highly practised skill in modern literate societies. The observation of a striking disorder of reading called pure alexia (PA) after damage to a region of left ventral occipito-temporal cortex, corresponding to the posterior fusiform gyrus (pFG), suggests that this region comes to specialise in rapid parallel processing of the familiar letter patterns that make up words (e.g., Vinckier et al., 2007). Others have instead focussed on the particular visual demands posed by reading (e.g., Behrmann & Plaut, 2013b), suggesting that left pFG is involved in processing items that require high acuity foveal vision, consistent with neuroimaging studies showing this region to be active not only for words but other complex visual stimuli such as faces. The goal of this paper was to provide a detailed examination of face processing abilities in a large sample of patients with damage to the left pFG and associated reading deficits of varying severity.

PA refers to a reading deficit that is apparent in the context of intact writing, normal spelling and no aphasia (Benson and Geschwind, 1969, Capitani et al., 2009). The reading performance is defined as pathologically slow, inefficient processing of letter strings across various transformations (e.g., font, size and case) with an exaggerated effect of word length on speed and/or accuracy of reading performance (Bub et al., 1993, Déjerine, 1892, Shallice and Saffran, 1986, Warrington and Shallice, 1980). In addition to effortful reading, these patients routinely use a sequential and sometimes explicit part-based (i.e., letter-by-letter) reading strategy to circumvent their inability to recognise whole words by boosting letter level activation. This contrasts with normal skilled adult reading, where letters are recognised in parallel with a negligible effect of word length on performance (Weekes, 1997). As these patients do not present with a frank visual object agnosia (at least when measured in terms of reduced accuracy: cf. Roberts et al., 2013), PA has been viewed by some as a reading-specific deficit (Arguin and Bub, 1993, Bub and Arguin, 1995, Howard, 1991, Saffran and Coslett, 1998, Warrington and Shallice, 1980, Yong et al., 2013). This is consistent with the purported specialisation of the left pFG region, sometimes called the “visual word form area” (VWFA: Cohen and Dehaene, 2004, Cohen et al., 2000, Cohen et al., 2004, Cohen et al., 2002, Dehaene and Cohen, 2011), for orthographic processing.

An alternative perspective on PA assumes that the inefficient reading is symptomatic of a visual processing deficit which reveals itself most readily with orthographic stimuli due to the intrinsically high demands they place on the visual system (Behrmann et al., 1998a, Behrmann and Plaut, 2013b, Behrmann et al., 1998b, Behrmann and Shallice, 1995, Farah and McClelland, 1991, Friedman and Alexander, 1984, Mycroft et al., 2009, Nestor et al., 2013, Roberts et al., 2010, Roberts et al., 2013, Starrfelt and Behrmann, 2011, Starrfelt and Gerlach, 2007, Starrfelt et al., 2010, Starrfelt et al., 2009). Efficient reading relies not only on the identification of component letters but also heavily on the accurate encoding of letter position and relative letter order. Neuroimaging results indicate that the VWFA is sensitive to the familiarity of subword letter combinations like bigrams and trigrams (Binder, et al., 2006, Vinckier et al., 2007). Visual processing deficits in PA could therefore undermine the rapid and accurate perception of the configuration of letter combinations that allow for identification of specific words.

It has been proposed that higher order visual processing areas are retinotopically organised, with a medial to lateral gradation of peripheral to foveal information across the ventral occipito-temporal cortex in both hemispheres (vOT; Hasson et al., 2003, Hasson et al., 2002, Levy et al., 2001, Malach et al., 2002). Visual acuity (sensitivity to high spatial frequencies) is highest in the fovea and drops toward the periphery (Fiset et al., 2006a, Fiset et al., 2006b, Starrfelt et al., 2009, Tadros et al., 2010, Tadros et al., 2013). Foveal vision is projected to the pFG and this region is maximally active for stimuli that require fine visual discrimination. This is in keeping with work demonstrating that (1) skilled readers show enhanced length effects when words are filtered to include only low spatial frequency information (Fiset et al., 2006a, Tadros et al., 2009), (2) patients with left pFG lesions show reduced sensitivity to medium to high spatial frequencies (Roberts et al., 2013; but see also: Starrfelt, Nielsen, Habekost, & Andersen, 2013) and (3) the left hemisphere becomes biased for high spatial frequency input over the course of development (Ossowski & Behrmann, 2015).

In line with evidence that non-language visual stimuli elicit activation in the VWFA (Behrmann and Plaut, 2013a, Behrmann and Plaut, 2013b, Price and Devlin, 2011, Price et al., 2006, Price et al., 2003, Vogel et al., 2012), the retinotopic account predicts that patients with left pFG damage should show processing deficits for all stimuli that require high acuity vision by virtue of their visual complexity and potential confusability. There is now a body of evidence demonstrating that PA patients are also impaired for visually complex non-linguistic stimuli when reaction times are considered as a measure of processing efficiency. An initial demonstration showed a group of five PA patients to be impaired in naming line drawings of familiar objects rated high in visual complexity (Behrmann et al., 1998a). Deficits in both object naming and object name-to-picture matching in patients with left pFG damage have more recently been found to be linked to the severity of the reading impairment as measured by the size of the length effect (Roberts et al., 2013). Processing unfamiliar non-linguistic symbols and checkerboard patterns has also been found to be impaired in letter-by-letter readers (Mycroft et al., 2009). Matching performance of patients with left pFG lesions on checkerboard stimuli and logographic characters is particularly impaired when these are both complex and presented with visually similar foils, and it is under these conditions that the strongest correlations with reading performance in terms of the size of the length effects emerge (Roberts et al., 2013).

Face recognition involves both feature identification and configural processing of various types (first-order feature arrangement, second-order feature spacing and gestalt holistic processing: Maurer, Grand, & Mondloch, 2002). Fluent reading is similar to face recognition in that it also involves both letter identification and various types of configural processing (letter position, relative letter order and global word shape processing). Indeed, a number of functional neuroimaging studies have found overlapping activations in left pFG for words and faces (Hasson et al., 2002, Kveraga et al., 2007, Mei et al., 2010, Vogel et al., 2012, Woodhead et al., 2011), with some even revealing overlap at the voxel level (Nestor et al., 2013). In addition, although face identification deficits are commonly associated with damage to the right pFG, including the fusiform face area (FFA), these are worse in cases of bilateral damage (Barton, 2008), indicating a contribution of left pFG as well (Mestry, Donnelly, Menneer, & McCarthy, 2012). We would therefore expect to see evidence of face processing deficits in patients with left pFG damage, despite the functional preservation of right hemisphere occipito-temporal regions implicated in face processing.

Indeed, a number of studies to date have reported cases in which patients with damage to the left fusiform have shown evidence of face processing deficits (Behrmann and Plaut, 2013a, Bub, 2006, Farah, 1991, Liu et al., 2011, Mestry et al., 2012). Behrmann and Plaut (2013a) used a discrimination task that involved different trials where the distractor had been morphed to the target to differing degrees, which affects feature-based and configural processing, and their four PA patients showed similar deficits to those of their three prosopagnosic patients with damage to the right pFG. In matching tasks involving changes over depth rotation and orientation, both thought to disrupt configural processing, both the PA and prosopagnosic patients were impaired. It is possible the impairment for PA patients arose due to disruption of basic featural processing, given this information is carried by the higher spatial frequencies (Hayes et al., 1986, de Heering and Maurer, 2013). At the same time, although it has been suggested that configural information is relatively preserved at lower spatial frequencies (Goffaux, Hault, Michel, Vuong, & Rossion, 2005), it is also the case that skilled adults are sensitive to very subtle second-order variations that are close to the limits of acuity (Haig, 1984, Maurer et al., 2002) and hence configural processing may well be disrupted in PA. Support for this notion is provided by functional imaging studies showing left pFG activation when processing faces that differ only in terms of second-order feature spacing (Rhodes, Michie, Hughes, & Byatt, 2009).

The mechanisms underpinning the face identification deficits in PA therefore remain unclear. This work aimed to examine face processing in a large sample of patients with left pFG damage and associated reading deficits of varying severity. We first explored whether nine patients showed deficits in familiar face identification in both expressive and receptive tasks. Although these patients do not present with prosopagnosia, they may well be impaired in their speed of identification, even for familiar faces that offer the opportunity for top-down support. We then assessed performance for 16 patients on a discrimination task involving novel faces that varied on feature identity, second-order spacing (by manipulation of internal distribution or external contour), or both. To the extent that letter identification can be preserved in PA (Behrmann & Plaut, 2013a), but that problems in the perception of the configuration of letters undermines fluent reading, we expected our patients with left pFG damage will show particular deficits for the second-order spacing conditions but relatively good performance for the feature identity condition. This prediction agrees with the finding that, in normal participants, more activation is seen for the spacing than featural condition in both right and left pFG, while higher activation for the featural than spacing condition is observed mainly in frontal regions (see Fig. 3 and Table 2, Maurer et al., 2007). If damage to left pFG undermines the configural processing both for words and faces, then we would further expect that novel face processing deficits would be linked to the severity of the reading disorder, both categorically and correlationally.

## 2 Method

### 2.1 Patients

**Fig. 1:**
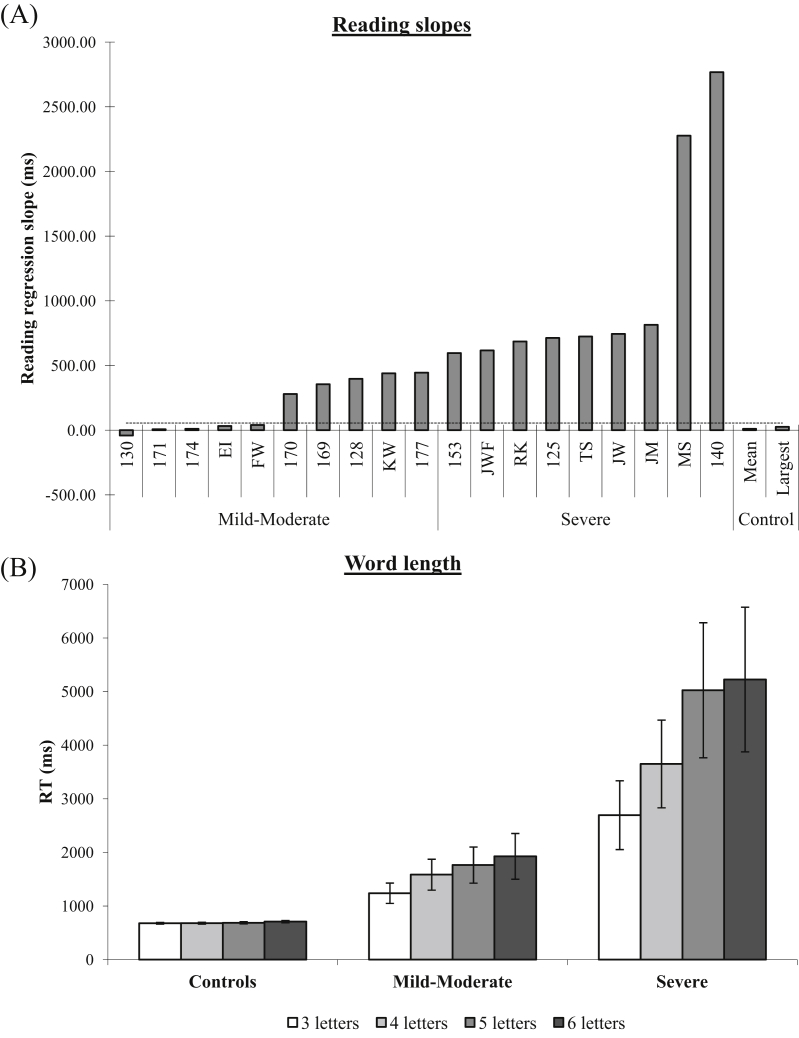
Summary reading data for the 19 patients included in the study for (A) the reading regression slope and (B) the mean reading speed as a function of word length. Error bars indicate ± standard error. Dashed line in (A) is control mean plus 2 standard deviations.

The cohort comprised of nine patients recruited from local NHS speech and language therapy services in the United Kingdom (UK) and a further 10 patients through collaboration with the University of Arizona (AZ). The study was approved by the local NRES committee in the UK and Institutional Review Board of the University of Arizona, and informed consent was obtained in all cases. To explore the impact of severity upon performance, it was necessary to recruit a broad range of patients using both behavioural and lesion criteria. Therefore, inclusion was based on neuroradiological evidence of damage to left ventral occipito-temporal cortex and/or a reading deficit characterised by an abnormally strong effect of length on reading speed. There was a range of severity among the recruited patients as measured by reading speed on a subset of the 3, 4, 5, and 6 letter word lists developed by Weekes (1997). For measuring correct RTs in tasks requiring a spoken response (e.g., reading, face identification), RTs were measured in the AZ patients using a voice key. For the (typically more severe) UK patients, RTs were established offline via a digital recording of each experimental trial using WavePad software (NCH, Swiftsound: www.nch.com.au/wavepad). The reading of a number of these UK patients was characterised by overt letter-by-letter identification of some letters in the string, and hence a voicekey would have produced inaccurate reaction times corresponding to identification of first letter. The waveforms of the sound files for each patient were inspected to derive a latency from the onset of stimulus presentation (indicated by a short 50 msec beep) to the onset of the correct reading response for that word. Given that PA is characterised by the abnormal length effect as well as slow reading times, we stratified our patients with left pFG damage according to the slope of their length effect, as computed over their average correct reaction times for 3, 4, 5 and 6 letter words (after Roberts et al., 2013). The results are shown in Fig. 1A (raw individual patient RT and accuracy data are provided in Supplementary Materials). The sample was split into two severity-based subgroups on the basis of the slope of their length effect in RT: a mild-moderate group of 10 patients and a severe group of nine patients. The average reading speed as a function of word length for each group is summarised in Fig. 1B.

### 2.2 Lesion mapping

**Fig. 2:**
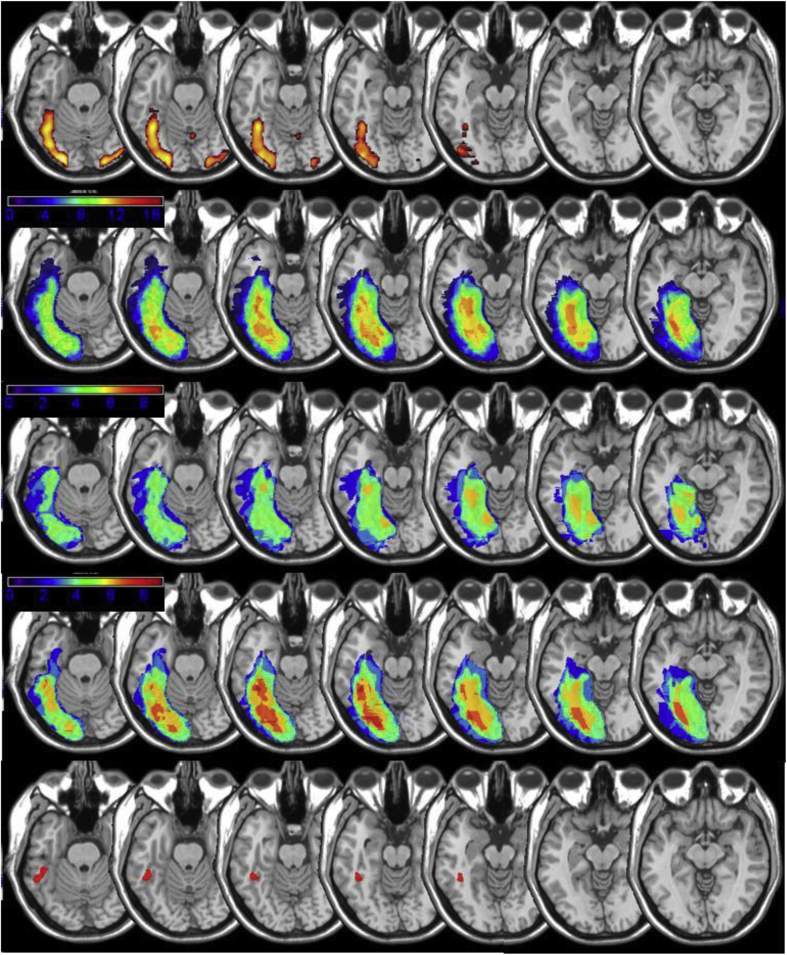
Row 1: fMRI activation during a reading task in 15 normal subjects (words – checkerboards, *p* < .05; FDR) Row 2: lesion overlap maps for all 17 patients included in the study with scans; Row 3: lesion overlap maps for the eight patients with the mildest reading impairment; Row 4: lesion overlap maps for the nine patients with the most severe impairment; and Row 5: Lesion map for patient 125, with a severe reading impairment, showing a small lesion confined to the left fusiform gyrus/occipito-temporal sulcus. .The axial slices of the MNI template brain in MRIcron have been rotated −15° from the AC-PC line in order to display the entire posterior-anterior course of the fusiform gyrus.

Lesions were reconstructed based on high-resolution research MRI or clinical MRI/computed tomography (CT) scans that were available for 17 of 19 participants (scans were unavailable for two UK patients, FW, KW). A lesion region of interest (ROI) was created for each patient using MRIcron software (http://www.cabiatl.com/mricro/mricron/). For research MRI scans, lesions were manually drawn directly on the patients' T1-weighted structural brain images at 1 mm intervals and then normalized to the standard MNI template brain using the lesion volume as a mask during the normalization process (Andersen et al., 2010, Brett et al., 2001). For the clinical CT and MRI scans, lesions were manually drawn onto the standard MNI template brain oriented to match the alignment of the scans (see Andersen et al., 2010, and Roberts et al., 2013 for additional details of our lesion mapping methods). Individual ROIs were subsequently combined to generate the lesion overlap maps. As can be seen in Fig. 2, most patients had damage to left pFG regions that show activation in normal subjects during a reading task. In two cases, imaging revealed additional damage to right medial occipital cortex, but in no cases did the lesions extend to right hemisphere ventral occipito-temporal regions implicated in face processing (i.e., the OFA/FFA). As can be seen in comparison of the lesion overlap maps in Rows 3 and 4 of Fig. 2, damage to the left pFG was more pronounced and consistent for the severe than the mild-moderate groups. Although lesions did extend beyond this region in some patients in both groups, this was not universally the case, and the bottom row of Fig. 2 presents the lesion map for patient 125, who had a relatively small lesion confined to the left fusiform gyrus/occipito-temporal sulcus in the presence of a severe reading impairment (see Fig. 1).

### 2.3 Background neuropsychological assessment

Each patient completed a battery of neuropsychological assessments to give a profile of their cognitive abilities. UK and AZ patients completed slightly different background tests (Table 1, Table 2, respectively). For UK patients, who comprised most of the severe subgroup, the Visual Object and Space Perception battery (VOSP; Warrington & James, 1991) was used to test a range of visual and visuospatial skills such as identifying incomplete letters and naming progressively more difficult silhouettes of common objects (for a detailed description of each task, see Warrington & James, 1991). A further battery of assessments explored semantic and phonological processing (see Roberts et al., 2013 for full details of these tests).

Semantic tasks were taken from the Cambridge Semantic Memory test battery (CSM; Adlam et al., 2010, Bozeat et al., 2000). The battery contains 64 items representing 3 subcategories of living things (animals, birds, and fruit) and 3 subcategories of artefacts (household items, tools, and vehicles) matched for psycholinguistic variables such as familiarity and age of acquisition. Knowledge of all items is assessed in verbal and non-verbal modalities of stimulus and/or response. The semantic memory tests administered include simple oral picture naming, word comprehension, and associative picture matching. For spoken word–picture matching (WPM), the participant is presented a spoken name and a picture array consisting of 10 items from the same category (e.g., birds); the task is to point to the item named by the examiner. Non-verbal associative knowledge is assessed by the Camel and Cactus Test (CCT), designed along the principles of the Pyramids and Palm Trees test (PPT; Howard & Patterson, 1992). Participants are required to choose one of four alternatives that has an associative relationship with the target item. An additional measure of verbal semantic knowledge, the synonym judgment test (Jefferies, Patterson, Jones, & Lambon Ralph, 2009) was also administered, which involved deciding which of three words was closest to a target word.

Phonological tasks included same–different phonological discrimination (PALPA 2; Kay, Lesser, & Coltheart, 1992), rhyme judgment (PALPA 15; Kay et al., 1992), and phonological segmentation and blending (Patterson & Marcel, 1992).

On the more visually challenging Silhouettes and Progressive Silhouettes tests of the VOSP, the majority of UK patients showed evidence of general visual processing deficits. Most patients were impaired in picture naming which is consistent with a visual deficit, although this could also reflect additional word finding difficulties. The more severe patients also showed mild but measureable impairments on some receptive semantic tests involving only a choice response. All patients had preserved working memory and were in the normal range on the minimal pairs test (PALPA 2) and the rhyme judgment test (PALPA 15). Performance was also excellent on the more demanding tests of phonological segmentation and blending, with the exception of patient RK (who suffered from significant age-related hearing loss).

Table 2 presents background neuropsychological data for the AZ patients who comprised most of the mild-moderate subgroup. Comparable tests were used between UK and AZ patients whenever possible (e.g., CCT UK = PPT AZ; CSM Naming UK = BNT AZ; analogous phonological processing tasks, etc.). Some patients showed mild impairments on orthographic letter matching and lexical decision tasks from the PALPA battery (Kay et al., 1992). Most patients were also impaired picture naming and/or semantic matching tasks, and indeed a picture naming impairment was the only abnormality seen for patient 125. All patients were in the normal range on rhyme judgment (bar patient 177), phoneme segmentation (although patient 169 scored 2 points below the normal cut-off), and minimal pair discrimination.

Inherent in large neuropsychological studies, not all patients could complete the full set of experimental tasks. This was due to further neurological events, demise, or medical illness. Nine patients completed the famous faces tasks, while 16 patients completed the Jane Faces task.

### 2.4 Spatial frequency sensitivity

**Fig. 3:**
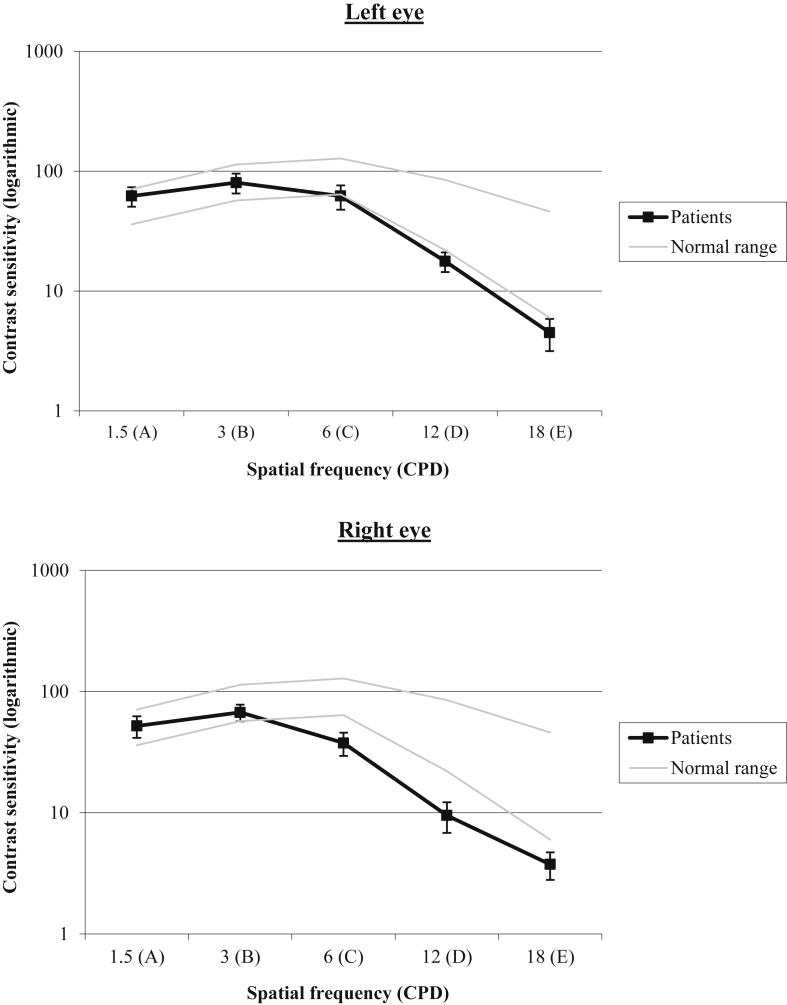
Functional Acuity Contrast Test results for eight of the nine UK patients in the current study. Grey lines represent normal range.

The retinotopic eccentricity account predicts that sensitivity to moderate to high spatial frequency should be impaired in patients with damage to the left pFG. To assess this we administered the functional acuity contrast test (http://www.stereooptical.com/) to eight of the nine UK patients (as reported in Roberts et al., 2013). The test evaluates sensitivity across a range of spatial frequencies and contrast. The test comprises a progression of high-quality, sine-wave gratings that probe sensitivity to 1.5, 3, 6, 12, and 18 cycles per degree. The contrast step between each grating patch is .15 log units. The contrast range spans the variation of contrast sensitivity found in the normal population. Following the standard instructions, the patients were asked to decide whether each grating was tilted right, vertical, or left. Fig. 3 displays average results from the patients. Contrast sensitivity would fall between the grey lines in 90% of the normal population, hence a functional impairment is indicated if the curve is below the normal range for either eye. All patients demonstrated abnormal contrast sensitivity profiles at the medium and high frequencies (at or below the control minimum at 12 to 18 cycles per degree, some at even 6 cycles per degree: see Supplementary Materials for individual data), which is a key frequency range for recognition of letters (Fiset, Arguin, et al., 2006), as well as objects (Roberts et al., 2013) and faces (Goffaux et al., 2011).

## 3 Identification of famous faces

Firstly, we explored whether these patients with left pFG lesions exhibited deficits in the speed or accuracy of identification of familiar faces, a characteristic of acquired prosopagnosia arising from lesions involving the right pFG (Damasio et al., 1982, Meadows, 1974). Both expressive (picture naming) and receptive (name-to-face matching) abilities were assessed in all the UK patients (EI, FW, KW, JWF, RK, TS, JW, JM, MS). AZ patients did not complete this task because the faces were specific to a British audience. Nine controls comparable to patients with respect to age and years of education also completed the task. All control participants had no previous history of neurological problems.

### 3.1 Materials

Images of famous faces were selected for this test if a high proportion of individuals rated the faces as “iconic” or “very famous”. Raters were participating in control testing at The University of Manchester, UK and were comparable to the patients with respect to age and years of education. Stimuli consisted of 40 greyscale photographs with an average width and height of 180 × 250 pixels, a horizontal and vertical resolution of 96 dpi and a colour pitch depth of 8.

### 3.2 Procedure

In this and subsequent tasks, stimulus presentation was controlled using E-prime software (Schneider, Eschman, & Zuccolotto, 2002). Face identification was probed with two tasks – naming and cross-modal (word-face) matching. The administration of each set of materials began with 16 practice trials, followed by the 40 experimental trials. For naming, stimuli were presented centrally following a fixation cross and the participants were asked to name them (e.g., “Marilyn Monroe”). In the matching task, participants were presented with a target name in both spoken (by the experimenter) and written (for an unlimited duration) form. When the participant was ready, this was followed by a backward pattern mask (in the same position of the stimuli, to avoid any visual persistence of the text) and a display of four face choices, one in each quadrant of the screen. For example, the name “Richard Branson” followed by a series of four faces: Donald Trump, Noel Edmonds, Richard Branson, Alexi Lalas. Targets were counterbalanced and distributed equally across the four positions across the trials. Stimuli remained on the screen until a response was given. Participants indicated their choice by means of a key press. RT and accuracy data were recorded. The order in which trials were presented in naming and matching tasks was identical for all participants. Participants completed the naming task first and then the matching task, at least 2 weeks apart. To determine if hemianopia had any effect on performance in these and subsequent experiments, left and right hemifield word reading and object naming was probed in a subset of five patients (FW, EI, JW, JM, MS). No significant difference between performance in accuracy or RT in each hemifield was present for reading or naming (see Supplementary Materials in Roberts et al., 2013 for details). We therefore do not expect visual field defects to exert a marked impact on face processing, at least with a single centrally presented stimulus.

### 3.3 Results

**Fig. 4:**
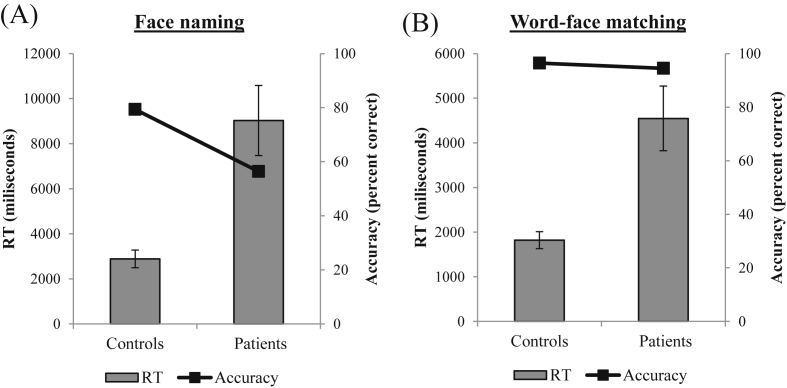
Means reaction times and accuracy for nine patients and nine matched controls for the famous face (A) naming (patient accuracy range = 15–93%) and (B) matching (patient accuracy range = 63–100%). Error bars indicate ± standard error.

Fig. 4 displays results for patient and control groups on naming (A) and word-face matching (B). Performance of the two groups (controls *vs* patients) was compared with independent samples *t*-tests. Relative to controls, patients had slower RTs [*t*(16) = −3.82, *p* < .001] and were less accurate [*t*(16) = −2.42, *p* < .05] for naming. Comparable *t*-tests for word-face matching revealed this was also the case in RT [*t*(16) = 3.63, *p* < .005] but not accuracy [*t*(16) = .85, *p* = .409]. Crawford's T statistic (Crawford, Garthwaite, & Porter, 2010) was used to determine which individual patients differed from controls for each task. These analyses revealed that the majority of patients (bar FW, JM for naming and EI, JM, TS for WPM) were impaired in relation to controls in accuracy, speed or both (see Supplementary Materials). Those patients who were unimpaired were mildest (EI, FW) and/or approaching significance on the Crawford statistic (*p* ≤ .10). These results are striking as the low accuracy of face naming in these cases is reminiscent (albeit milder in form) of that seen in prosopagnosic patients with right pFG lesions (Behrmann & Plaut, 2013a). The persistence of deficits in the matching tasks indicates that these face identification deficits were not the result of more general word finding difficulties.

## 4 Discrimination of novel faces

As predicted, the patients as a group were clearly impaired at identification of familiar famous faces. This would not have been so apparent if accuracy measures alone had been used. Instead, the deficit is primarily reflected in speed, particularly in the receptive task. However, the degree of impairment may be underestimated using familiar faces because intact top-down semantic information might boost impaired early processing, as has been suggested in the case of word processing (e.g., Roberts et al., 2010). We therefore sought to extend these findings using novel faces that have no intrinsic meaning or familiarity. In addition, the use of novel faces has the advantage that stimuli can designed to assess the use of feature identity versus second-order spacing information (both of internal features and also relative to the external contour). In this experiment, therefore, we used the Jane Faces task (Maurer et al., 2007, Mondloch et al., 2002) to explore the mechanisms for deficits in novel face processing in patients with a left pFG lesion. We tested 16 patients on this task and to assess the impact of severity, they were divided into two equal groups on the basis of their length effect in reading aloud, with the mild-moderate group consisting of 130, 171, 174, 170, 169, 128, KW, 177 and the severe group consisting of 153, JWF, RK, 125, JW, JM, MS, 140. We also explored the extent to which severity of the reading deficit predicted face discrimination performance using a correlational approach. The task was also completed by a control group (*N* = 15) who were comparable to the patients with respect to age and years of education. All control participants had no previous history of neurological problems.

### 4.1 Materials

**Fig. 5:**
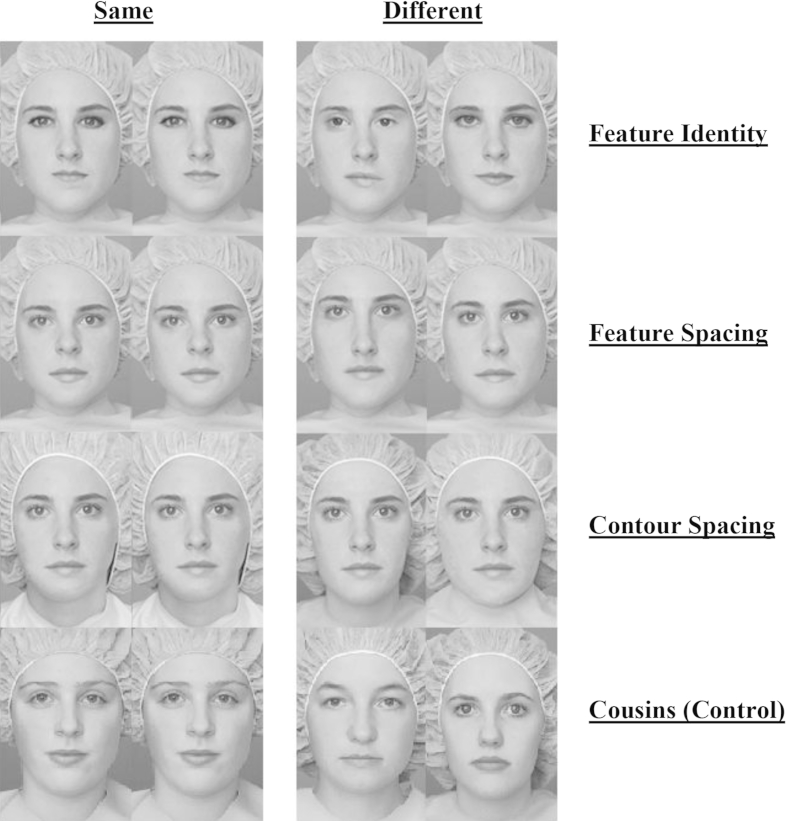
Examples for same and different stimuli for each condition of the Jane Faces task.

The stimuli used have been reported elsewhere (Mondloch et al., 2002). To summarise, a grayscale photograph of a single face (called “Jane”) was modified and three sets of face stimuli (feature identity, feature spacing and contour spacing – see Fig. 5) were created to create twelve new versions (“Jane's sisters”). To tap featural processing, four modified faces in the feature-identity set were created by replacing either Jane's eyes, mouth, or both with the features of the same length from different females. Such modifications have insignificant effects on second-order processing because the size and location of individual features remain constant. To tap second-order processing, four modified faces in the feature-spacing set were created by adjusting the spacing between the eyes up or down from the original, the eyes closer together or farther apart, and the mouth up or down. This modification covered variations in spacing among adult female faces in the population, without being so large that the faces appeared malformed or unnatural (Farkas, 1981). The four modified faces in the contour-spacing set were created by adjusting the external contour, pasting the internal portion of the original face within the outer contour of four different females. This modification changes the frame of the face and hence necessarily also the spacing between features and the external contour (e.g., spacing from the bottom of the mouth to the chin contour). Both the feature-spacing and contour-spacing modifications have negligible effects on information about local features. The control “cousin” stimuli consisted of Jane and three different female faces, hence varied on all dimensions. All stimuli were 10.2 cm wide and 15.2 cm high (5.7° × 9.1° from the testing distance of 100 cm).

### 4.2 Procedure

Participants were asked to make visual discriminations between two faces presented simultaneously side by side centred on the screen (see Fig. 5 for examples). Each participant was instructed to press a key to indicate if the faces looked the same or different. The experimenter initiated the experiment by saying: “This is Jane (the original model was presented on the screen), Jane has 12 sisters that look a lot like her (the twelve modified versions of Jane were shown). See how they all look alike, like twins? Well, now we are going to play a game to see if you can tell apart these sisters. You will see two faces. They may be different sisters, or it may be the same sister twice. Your job is to indicate whether the two faces are the same or different. Press “f” for same and “j” for different. Try to be as accurate but as quick as possible.” The instructions for the key press were then repeated and participants were asked to demonstrate what they should do if they saw pairs of the same or different faces.

Each trial was initiated automatically after the participant indicated his or her readiness to start the experiment. A fixation cross was presented for 500 msec before being replaced by the target face pairs. Stimuli remained on the screen until a response was given. All participants were tested on 90 trials divided into three 30-trial blocks: feature identity, feature spacing, and contour spacing. In each block, 15 trials involved presentation of the same face and 15 trials involved the presentation of different faces. Trials were blocked to encourage participants to use specific processing strategies (Yovel & Duchaine, 2006). Prior to the experimental blocks the participant was given six practice trials, one same and one different trial from each stimulus set with words of encouragement provided as feedback.

The order in which blocks were presented was the same for all participants (feature spacing, feature identity, contour spacing, cousins) (Mondloch et al., 2002). Within each block, each face was presented half of the time on a “same” trial and half of the time on a “different” trial. All participants saw the same random order of trials in each block. After the third block, a block of trials with Jane's cousins were presented. The experimenter initiated this block by saying “Great job! Now we're going to play a game with Jane and her cousins. This time, none of her sisters will show up. It's just Jane and her cousins. Just like before, you'll see two faces in a row, and your job is to press “f” if you think the faces were of the same person, and “j” if you think they were different. Are you ready?” This cousins block consisted of 32 trials with either the same face twice (16 trials) or two completely different faces the necessarily differed on features, spacing and contour (16 trials). The task lasted for around 30 min. See Fig. 5 for examples of the stimuli used for each of the conditions.

### 4.3 Results

The average RT and accuracy of patients and controls are provided in Table 3, Table 4 respectively (see Supplementary Materials for individual data). Repeated-measures ANOVA was conducted on RT and accuracy with severity (controls/mild-moderate/severe) as a between-subject factor and condition (feature identity/feature spacing/contour spacing/cousin control) as within-subject factors. Greenhouse-Geisser corrected values are provided in order to compensate for any violations of sphericity. The results for RT revealed a significant main effect of severity [*F*(2, 28) = 13.94, *p* ≤ .0001], condition [*F*(2.37, 66.32) = 33.73, *p* < .0001], but no interaction between the two [*F*(4.74, 66.32) = 1.39, *p* = .24]. The results for accuracy revealed no effect of severity [*F*(2, 28) = 1.97, *p* = .16], a significant main effect of condition [*F*(2.21, 61.82) = 50.67, *p* < .0001], but no interaction between the two [*F*(4.42, 61.82) = .52, *p* = .74].

Considering RT performance for patient 125, with a severe reading impairment and a small lesion constrained to the left pFG, the feature identity condition was significantly slower than that of the control group (*z* = 7.29, *p* < .0001, one-tailed), as was the feature spacing (*z* = 5.75, *p* < .0001, one-tailed), contour spacing (*z* = 3.51, *p* = .006, one-tailed), and cousins (*z* = −2.71, *p* = .003) conditions. Patient 125 was less accurate than controls in the feature identity (*z* = −1.86, *p* = .03, one-tailed) and cousins (*z* = −2.11, *p* = .02) conditions but accuracy on the feature spacing (*z* = −.88, *p* = .20, one-tailed) and contour spacing (*z* = −.31, *p* = .38, one-tailed) conditions fell within the normal range.

**Fig. 6:**
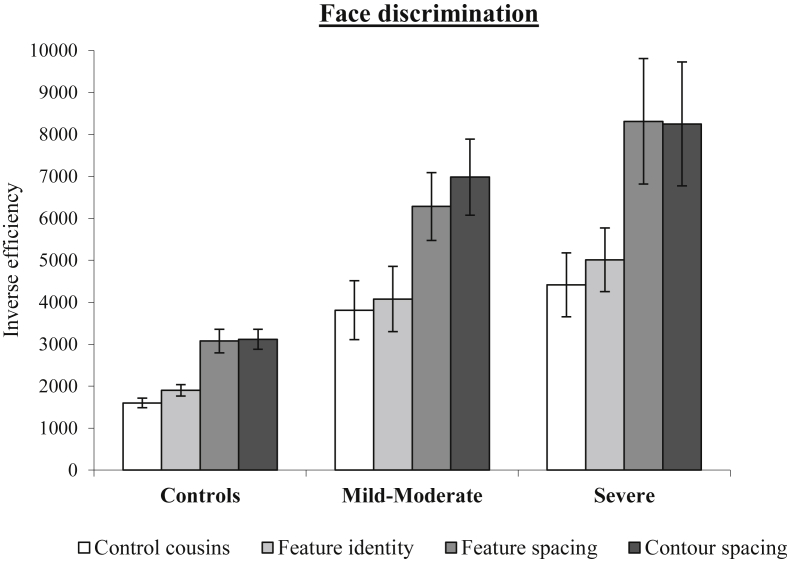
Performance for conditions of the face discrimination task for the patient subgroups split by severity (slope of the length effect in RT) and controls. Error bars represent standard error.

Inspection of Table 3, Table 4 indicates that there appear to be some trade-off between speed and accuracy that differ across severity groups. In order to more effectively compare the results over groups, we computed an inverse efficiency measure (Roberts et al., 2010, Roder et al., 2007). This is derived by dividing the mean correct RT for each condition by the proportion correct, producing a measure comparable to reaction time but corrected for variations in accuracy (see Supplementary Materials for individual data). Repeated-measures ANOVA (Greenhouse-Geisser corrected) on inverse efficiency values revealed significant main effects of severity [*F*(2, 28) = 15.17, *p* < .0001], condition [*F*(2.46, 68.76) = 41.21, *p* < .0001], and an interaction between the two [*F*(4.91, 68.76) = 3.27, *p* = .01]. The form of the interaction can be seen in Fig. 6, which shows that poor patient performance is most pronounced for the second-order configural conditions involving changes in feature spacing or contour spacing, and somewhat more so for the more severe patients. The difference between the cousins and feature-identity condition was equivalent across all groups [*t*(21) = .06; *t*(14) = .36; ps > .115]. The difference between the cousins and feature-spacing condition was marginally significantly larger for the mild-moderate patients than controls [*t*(21) = 1.81 *p* = .085], but did not differ for the mild-moderate and severe patients [*t*(14) = 1.20; *p* = .252]. Similarly, the difference between the cousins and contour-spacing condition was significantly larger for the mild-moderate patients than controls [*t*(21) = 2.87 *p* = .0009] but did not differ for the mild-moderate and severe patients [*t*(14) = 624; *p* = .543]. Hence, these patients with left pFG damage and reading deficits seemed to show a more marked impairment for the spacing conditions requiring second-order processing relative to the feature-identity condition requiring first-order processing in this task.

Returning to the performance of patient 125, we can see the same form of interaction in inverse efficiency scores. The non-parametric Crawford Revised Standardized Difference Test (RSDT: Crawford & Garthwaite, 2005) revealed that the difference between the cousins and feature-identity condition for patient 125 was similar to that of controls [*t*(14) = .11, *p* = .45]. The difference between the cousins and feature-spacing condition was significantly larger for patient 125 than controls [*t*(14) = 2.03, *p* < .05, one-tailed], as was the difference between the cousins and contour-spacing condition [*t*(14) = 3.50, *p* < .002, one-tailed]. These results demonstrate a stronger impairment of processing in the spacing conditions than the feature-identity condition in a patient with a small lesion confined to the left pFG and a severe reading deficit.

To explore the relationship between reading behaviour and face discrimination, correlations were computed between the slope of the length effect in reading RT (as shown in Fig. 1A) and the inverse efficiency scores on each condition of the discrimination task. Spearman's correlations are presented in order to account for the possibility of nonlinear relationships. The slope of the length effect was significantly related to performance in the feature-identity condition (*r* = .45, *p* = .04), but not to performance in any other condition (*r*_s_ < .31, *p*_s_ > .23). This result suggests that the part-based processing strategy used by the patients to support their reading was useful in maintaining good performance in the conditions where faces differed only in the identity of component features, but did not help when it came to conditions that varied in terms of their second-order spacing relations.

Lastly, we considered whether variations in lesion size contributed to our results. Lesion volume was not significantly correlated with the slope of the length effect (*r* = .22, *p* = .21, one tailed). Lesion volume showed a significant negative correlation with the feature-spacing condition (*r* = −.49, *p* = .03), such that patients with larger lesions actually performed better. Lesion volume was not correlated with performance in any other condition of the face discrimination task (r_s_ > −.36; *p*_s_ > .10). This pattern of correlations indicates that the stronger reading and face processing deficits we observed for the more severe patients are not simply a consequence of variation in lesion extent.

## 5 Discussion

This research has demonstrated striking deficits in processing both familiar and novel faces in large sample of patients with damage to the left pFG, an area traditionally associated with written word recognition. Nine patients were clearly impaired in the identification of famous faces in both receptive and expressive tasks. Sixteen patients showed impairments in novel face discrimination that were particularly pronounced when this required sensitivity to second-order configural relations. These results are consistent with a retinotopic perspective on ventral occipito-temporal cortex such that the pFG regions of either hemisphere specialise in processing high acuity foveal input that is particularly important when processing complex and highly-confusable visual stimuli. Letter strings are heavily reliant on such processing, and indeed, these patients show deficits in terms of slowed reading and exaggerated length effects. A number of investigations have also revealed deficits in the processing of complex familiar and novel objects, and the extent of these impairments is linked to the severity of the reading disorder (e.g., Behrmann et al., 1998a, Cumming et al., 2006, Mycroft et al., 2009). This work extends initial observations of face processing deficits in patients with left pFG lesions (e.g., Behrmann and Plaut, 2013a, Mestry et al., 2012, Roberts et al., 2013) by establishing that these deficits extend across familiar and novel stimuli, and relate to the visual processing requirements of the novel faces in terms of the involvement of featural and configural processing.

In keeping with a retinotopic account, all eight of the UK patients in this study that were tested on the Functional Acuity Contrast Test showed diminished sensitivity to higher spatial frequencies (Roberts et al., 2013) in the context of damage to the pFG and reading problems. This is consistent with peak overlap of the patients' lesions in the left pFG region shown to be more active for processing gratings of high relative to low spatial frequency (Iidaka et al., 2004, Vuilleumier et al., 2004, Woodhead et al., 2011). In terms of the basis for the patients' problems discriminating between novel faces, we might have expected to observe stronger deficits in feature-identity processing, which has been suggested to be carried by the higher spatial frequencies, than second-order configural processing, for which lower spatial frequencies have been implicated as being crucial (e.g., Goffaux et al., 2005). In fact, we found the opposite pattern: relatively good discrimination on the basis of feature identity and relatively poor performance in the feature-spacing and contour-spacing conditions. The results for patient 125, with a severe reading deficit and marked impairment in the feature-spacing condition in the presence of a small lesion centred on the left pFG confirm the importance of this specific area in both reading and face processing, in line with functional imaging studies showing overlapping activations for words and faces in this region (Hasson et al., 2002, Kveraga et al., 2007, Mei et al., 2010, Vogel et al., 2012, Woodhead et al., 2011).

Given the lesion overlap methodology used here, we cannot be certain that deficits seen in other patients arose from damage to the same region as that implicated in patient 125. Lesions for many patients also encompassed primary visual processing areas (V1), and this is apparent in the prevalence of hemianopia across patients. We would argue, however, that these lower level visual problems did not underpin the patients reading and face processing deficits, as hemianopia was actually less prevalent in the severe (two patients with intact visual fields) than the mild-moderate group (one patient with intact visual fields). Moreover, it has been shown that the behavioural profile associated with hemianopic alexia does not entail he significant increase in length effects that characterised the reading of patients in our severe group (Leff, Spitsyna, Plant, & Wise, 2006). An additional caveat to the lesion overlap approach is that we cannot rule out the possibility that the lesion has resulted in cortical thinning of connected areas (Duering et al., 2012). Yet damage to the left pFG has consistently been associated with PA, and more recently with face processing deficits (e.g., Behrmann et al., 2013a), and the same region is active in normal participants during reading and face processing tasks (e.g., Woodhead et al., 2011). It therefore seems unlikely that damage to areas remote from the lesion made a significant contribution to the behavioural deficits we observed in our patients.

As the feature-spacing and contour-spacing conditions of the face discrimination task also proved to be the most difficult for healthy controls, it might be argued that the deficits seen in these conditions amongst the patients reflect a more general cognitive impairment that is only manifest under more demanding task conditions. Yet the deficits we observed for patients in familiar face identification tasks, which are minimally demanding for healthy control participants, imply that the patients were impaired specifically in face processing, most notably when this requires sensitivity to the relationships between component features. We therefore suggest that the deficits we observed for second-order conditions indicate a role for higher spatial frequencies in configural face processing. Indeed high acuity foveal vision is likely to be needed in order to detect subtle variations in spacing like those used in the present study. This proposal is supported by the results of functional imaging studies that have considered performance when processing faces differing only in second-order spacing and have found activation in both the right and the left pFG (Maurer et al., 2007, Rhodes et al., 2009), and studies that have observed higher activation in the left pFG when viewing faces composed of higher spatial frequency information (Iidaka et al., 2004, Vuilleumier et al., 2004).

While reduced sensitivity to higher spatial frequencies may well have undermined face identification and discrimination by impinging upon configural processing, this does not account for the surprisingly good performance seen in the patients when only featural processing was required. One possibility is that this was supported by coarser visual differences between faces in the feature-identity condition, such as contrast (Yovel & Duchaine, 2006). This interpretation seems unlikely, however, given that it was specifically performance in the feature-identity condition that correlated with the severity of the reading deficit. Instead, this correlation suggests that patients could efficiently discriminate based on changes in feature identity using a sequential feature analysis strategy analogous to the letter-by-letter behaviour seen when reading. The observation that the feature-identity condition did not elicit more activation than the feature-spacing condition in the left pFG of normal participants (Maurer et al., 2007), but did in regions like the left middle frontal gyrus (MFG), suggests that feature-identity discrimination as measured in this task may be a strategic process. This is consistent with functional imaging indicating a role for these frontal regions in sequential working memory tasks (Braver, Gray, & Burgess, 2007) and executively demanding processes (Duncan, 2010). As our patients had intact left frontal structures and working memory, it is possible that these systems allowed them to adopt an effective part-based strategy to compensate for diminished high spatial frequency sensitivity due to left pFG damage. This strategy can partially support reading of letter strings and permit face discrimination when it can be based purely on feature identity. This interpretation would require further investigation using functional imaging of patients with left pFG damage but it is consistent with the observation that activation of left MFG increased in a PA patient as their proficiency in application of the letter-by-letter reading strategy improved over time (Henry et al., 2005).

Our interpretation of preserved performance in the feature-identity condition by our patients with left pFG lesions does not imply that they have entirely intact and efficient feature-based processing of words or faces. Indeed, many patients with PA are impaired in speeded letter matching and letter identification tasks and some also misidentify letters when reading aloud (Cumming et al., 2006, Starrfelt et al., 2009, Woollams, et al., 2014). Hence it is not that these patients adopt a part-based strategy because their feature processing is normal, but rather, this approach helps to offset the impact of diminished sensitivity to high spatial frequency on parallel/configural processing (Fiset et al., 2006a, Tadros et al., 2010, Tadros et al., 2013). In the context of the novel faces task used here, with simultaneous presentation of choices and unlimited exposure duration, the part-based strategy was sufficient to support normal performance. This result, when combined with neuroimaging data showing left MFG activation for the feature-identity condition, suggests that normal participants also adopt a similar part-based strategy in this task. The presentation technique used here was adopted as pilot testing revealed the AZ patients with left pFG damage to be at chance with the brief exposure durations and sequential presentation originally used in this task (Mondloch et al., 2002). We are therefore of the view that configural and feature-based processing are both impaired following left pFG damage, presumably as a result of inefficient coding of high spatial frequency information, but the deficit is more pronounced for the former than the latter.

The results of the novel face discrimination task therefore suggest that high spatial frequency information is more critical for configural processing of complex visual objects (both faces and words) than for part-based processing of these same stimuli (i.e., letter-by-letter reading for words and feature-by-feature discrimination for faces). The disproportionate impairment of parallel/configural visual processing for both words and faces following damage to left pFG leads to compensatory reliance on a relatively preserved part-based strategy. Prosopagnosic patients with right pFG damage also seem to process faces by relying on a piecemeal or feature-based strategy (Van Belle et al., 2010), similar to our patients with left pFG lesions. It would seem that efficient parallel/configural processing of complex visual stimuli requires the functional integrity of both left and right pFG, whereas part-based processing can be supported by either hemisphere. Yet despite the similarities between PA and prosopagnosic patients in processing of words and faces, their performance is not identical. Behrmann and Plaut (2013a) found the length effects in word recognition to be more pronounced in PA than prosopagnosia, and conversely, the face processing deficits were more pronounced in prosopagnosia than PA. In addition, it was only the prosopagnosic cases who showed a reversal of the standard superiority of upright over inverted faces, with the PA patients showing an exaggeration of the normal pattern. These differences between PA and prosopagnosic patients indicate some degree of graded specialisation across the left and right pFG.

Although the retinotopic view does propose a broadly mirror symmetric organisation of the fusiform gyri (Malach et al., 2002), this is not to that deny some relative differences according to laterality do exist (Behrmann & Plaut, 2013b). These differences may stem from at least two factors. The first is the nature of frequency sensitivity. While there is evidence for the use of both low and high spatial frequency information over time across left and right pFG (Goffaux et al., 2011), there is nevertheless a degree to which the left pFG is relatively more sensitive to higher spatial frequency information while the right pFG is relatively more activated by lower spatial frequencies (Ossowski and Behrmann, 2015, Woodhead et al., 2011). The second difference between the left and right pFG relates to their connectivity, as their location means that they are likely to be more strongly linked to areas involved in linguistic versus person knowledge, respectively (Epelbaum et al., 2008, Lambon Ralph et al., 2001, Nestor et al., 2011, Pyles et al., 2013, Wang et al., 2011). Future comparative case series will be required to determine whether differences between word and face processing impairments in PA and prosopagnosia arise from variations in spatial frequency sensitivity and/or connectivity across the left and right pFG.

## Figures and Tables

**Table 1:** Demographic and background neuropsychological assessment for the 9 UK patients ordered, left to right, according to the severity of the reading impairment (slope of the length effect).

	Max.	Normal cut-off	EI	FW	KW	JWF	RK	TS	JW	JM	MS
**Demographics**											
Age	–	–	40	80	44	54	63	57	59	67	70
Sex	–	–	F	M	M	F	M	M	M	M	F
Handedness			RH	RH	RH	LH	RH	RH	RH	RH	LH
Years of education	–	–	13	11	10	10	10	10	11	10	10
**Lesion aetiology**			Stroke	Stroke	Stroke	Stroke	Stroke	Tumour resection	Stroke	Tumour resection	Stroke
**Lesion volume (cc)**			12.11	No scan	No scan	92.89	39.93	162.69	93.27	14.34	99.34
**Visual field loss**			RUQ	RHH	RHH	RHH	RHH	RHH	RHH	RUQ	RHH
**Working memory**											
Digit span											
Forward (12)	–	5	9	8	8	6	NT	8	7	12	10
Backward (12)	–	2	5	4	7	5	NT	4	4	7	6
**Visual processing**											
VOSP											
Incomplete letters	20	16	20	17	20	17	20	19	19	20	16
Silhouettes	30	15	21	21	19	24	20	22	25	18	19
Object decision	20	14	19	17	20	19	15	18	17	17	16
Progressive silhouettes	20	15	**11**	**14**	16	**8**	20	**5**	**8**	**11**	**9**
Dot counting	10	8	10	**7**	9	10	10	10	10	10	9
position discrimination	20	18	20	19	20	**16**	20	18	20	20	19
Number location	10	7	9	10	10	8	9	10	10	10	10
Cube analysis	10	6	10	9	**4**	10	6	10	9	10	7
**Semantic processing**											
Naming^a^	64	62	62	62	**58**	**56**	**56**	**41**	**59**	**61**	**45**
Camel and Cactus (pictures)^a^	64	52	61	59	**44**	61	52	**24**	52	61	**47**
Word-picture matching^a^	64	62	64	64	NT	NT	NT	63	64	63	62
96 Synonyms^b^	96	90	91	96	**74**	94	90	**83**	93	93	**81**
**Phonological processing**											
PALPA 2: Phonological judgement											
Total	72	64	68	71	71	72	72	68	71	72	71
Same	36	34	32	35	35	36	36	36	36	36	36
Different	36	30	36	36	36	36	36	32	35	36	35
PALPA 15: Rhyme judgement	60	43	47	57	59	58	57	56	57	56	53
Phoneme segmentation^c^											
Total	96	76	94	96	87	96	**73**	87	96	94	91
Addition	48	39	46	48	40	48	**36**	48	48	46	45
Subtraction	48	37	48	48	47	48	37	39	48	48	46

*Note*. **Bold** denotes abnormal performance. VOSP: Visual Object and Space Perception battery. pALPA: Psycholinguistic Assessment of Language Processing in Aphasia (Kay et al., 1992). NT: Not tested; RHH: right homonymous hemianopia; RUQ: right upper quadrantanopia; NFD: no field deficit.

a Bozeat et al. (2000).

b Jefferies et al. (2009).

c Patterson and Marcel (1992).

**Table 2:** Demographic and background neuropsychological assessment for the 10 AZ patients ordered left to right, according to the severity of the reading impairment (slope of the length effect).

	Max.	Normal cut-off	130	171	174	170	169	128	177	153	125	140
**Demographics**												
Age	–	–	80	78	63	60	72	54	62	69	65	67
Sex	–	–	M	M	M	M	M	M	M	M	M	F
Handedness	–	–	R	R	R	R	R	R	L	R	R	R
Years of education	–	–	18	14	18	14	14	18	10	11	12	10
**Lesion aetiology**	–	–	Stroke	Stroke	Stroke	Stroke	Stroke	Stroke	Stroke	Stroke	Stroke	Stroke
**Lesion volume (cc)**			37.23	38.33	5.15	56.82	74.42	97.69	51.91	42.11	2.19	50.96
**Visual field loss**			NFD	RUQ	RHH	RUQ	RHH^#^	RUQ	RUQ	NFD	NFD	RHH
**Working memory**												
Digit span forward	12	5	9	10	10	11	6	10	5	9	7	NT
**Visual/orthographic processing**												
Letter case matching (PALPA 19, 20)	52	51	52	51	52	52	**50**	52	52	52	52	See^a^
Letter discrimination in words/nonwords (PALPA 21)	30	27	30	30	28	29	28	28	**25**	28	29	100%^b^
Visual lexical decision (PALPA 25)	60	58	58	59	60	58	**48**	59	**38**	**37**	51	**47**
**Semantic processing**												
BNT	60	53	**32**	58	58	**46**	**42**	57	**39**	55	**43**	**30**
PPT (pictures)	52	49	**48**	51	52	52	51	52	**47**	50	51	**44**
Word-picture matching (PALPA 48)	40	39	40	40	39	39	39	40	39	40	40	100%^c^
Auditory synonym judgment (PALPA 49)	20	19	20	19	20	20	**17**	20	19	20	20	NT
**Phonological processing**												
Rhyme judgment	40	36	39	39	40	40	37	39	**33**	38	39	100^d^
Phoneme segmentation	80	71	71	78	79	79	**69**	80	**56**	77	79	See above
Minimal pair discrimination	40	38	39	40	38	40	40	40	**36**	39	40	See above

*Note*. **Bold** denotes abnormal performance. pALPA: Psycholinguistic Assessment of Language Processing in Aphasia (Kay et al., 1992); BNT: Boston Naming Test (Kaplan, Goodglass, & Weintraub, 1983); pPT: Pyramids and Palm Trees test (Howard & Patterson, 1992). NT: not tested; RHH: right homonymous hemianopia; RUQ: right upper quadrantanopia; NFD: no field deficit. **#** In addition to extensive left occipito-temporal damage, CT scan in this patient also indicated a right dorsomedial occipital lesion that was associated with a left inferior quadrant visual field defect.

a PALPA 18 (correct/reversed letter identification): 34/36, PALPA 22 (letter naming): 25/26 (lower), 26/26 (upper), upper-lower case conversion: 22/26; Western Aphasia Battery (Kertesz, 2006) Supplemental Subtests.

b Letter discrimination.

c Written word-picture/object matching.

d Repetition (words of increasing length, phrases, and sentences).

**Table 3:** Reaction times (and standard deviations) for the Jane faces task used in Experiment 2 according to condition and participant type. Patient 125 has a lesion constrained to left pFG and a severe reading deficit.

	Feature identity	Feature spacing	Contour spacing	Cousins (control)
Controls	1766 (519)	2246 (816)	2419 (978)	1477 (333)
Mild-Moderate	3306 (1093)	4062 (1375)	4384 (1369)	3140 (1424)
Severe	4621 (1951)	5528 (2563)	5688 (2616)	3952 (1905)
Patient 125	5550	6936	5860	4330

**Table 4:** Percentage accuracy (and standard deviations) for the Jane faces task used in Experiment 2 according to condition and participant type.

	Feature identity	Feature spacing	Contour spacing	Cousins (control)
Controls	93.11 (6.95)	74.89 (16.52)	76.67 (12.79)	93.96 (9.18)
Mild-Moderate	87.5 (16.31)	67.5 (16.11)	65 (13.8)	85.32 (14.15)
Severe	92.5 (6.61)	67.08 (12.01)	70.42 (10.61)	90.11 (9.9)
Patient 125	80	60	73.33	75
